# Optimizing Revision Lumbar Surgery: The Unilateral Biportal Endoscopic Approach

**DOI:** 10.7759/cureus.98707

**Published:** 2025-12-08

**Authors:** Alhareth Maaya, Jin Hwa Eum

**Affiliations:** 1 Neurosurgery, Ain Al Khaleej Hospital, Al Ain, ARE

**Keywords:** minimally invasive and robotic spine surgery, revision of surgery, spinal cord, unilateral biportal endoscopic, visual analog scale

## Abstract

Background

Revision lumbar surgery is technically challenging due to postoperative scarring, altered anatomy, and high complication risks. Conventional open revisions cause more tissue damage and slower recovery. The Unilateral Biportal Endoscopic (UBE) technique, offering improved visualization and maneuverability, may enhance safety and outcomes in revision procedures.

Objective

This study aimed to evaluate the efficacy and safety of the UBE technique in managing complex lumbar revision surgeries.

Methodology

A retrospective analysis was conducted on 10 patients who underwent revision lumbar surgery using the UBE technique between February 2022 and February 2024. Clinical outcomes were assessed using the Visual Analog Scale (VAS) for pain and the Oswestry Disability Index (ODI). Complications and patient satisfaction were also evaluated.

Results

Significant reductions in pain and disability were observed postoperatively. The mean preoperative VAS scores for leg and back pain decreased from 8.1 to 0.8 and from 7.5 to 1.7, respectively (p < 0.001). The mean ODI score improved from 67.5 to 17.5 (p < 0.001). No major complications occurred, and 70% of patients reported excellent satisfaction.

Conclusion

UBE is a safe and effective minimally invasive approach for complex lumbar revision surgeries. It provides substantial pain relief, functional recovery, and low complication rates. By preserving soft tissues and offering superior visualization, UBE shows promise as an advanced alternative to conventional revision techniques.

## Introduction

Revision lumbar spine surgery is a complex procedure indicated for recurrent pain, instability, hardware failure, or adjacent segment disease after initial surgery [1]. These procedures are complicated by postoperative fibrosis, distorted anatomy, and increased risks of dural tears, infection, and poor functional recovery. Conventional open revision techniques, while effective, are associated with significant soft tissue disruption, prolonged hospitalization, and suboptimal patient satisfaction [2]. There has been a paradigm shift toward minimally invasive techniques that minimize collateral damage while achieving comparable decompression and stabilization [3].

The Unilateral Biportal Endoscopic (UBE) approach has gained attention as an advanced minimally invasive technique that combines endoscopic visualization with the use of standard surgical instruments [3]. UBE uses two small incisions, one for the endoscope and one for instruments, offering advantages over open and other minimally invasive techniques, such as less muscle dissection, reduced blood loss, shorter hospital stays, and quicker recovery [4].

Biportal endoscopy, though relatively new, has shown promising outcomes in spinal decompressions and discectomies. Its use in revision surgeries is less studied, but UBE's enhanced visualization and precision make it well-suited for the complex challenges of revision lumbar cases, allowing safe navigation through scar tissue and altered anatomy while minimizing further damage [5]. Initially limited by cost and steep learning curves, minimally invasive spine surgery is now widely accepted. UBE, with its dual portals for better triangulation and precision, helps overcome earlier limitations and is particularly useful in complex revision cases [6].

Revision lumbar surgeries often yield poorer outcomes than primary procedures, with higher complication rates and less symptom relief. This is largely due to scar tissue, fibrosis, and risks like dural tears or nerve injury, along with the added complexity of addressing both the original pathology and new complications [7]. While UBE has been extensively investigated for primary lumbar decompression and discectomy, evidence supporting its application in revision lumbar surgeries remains limited. Most available literature focuses on primary degenerative conditions, leaving a critical gap regarding UBE's safety, feasibility, and long-term outcomes in the revision setting [8]. Given that revision cases carry higher complication rates and poorer functional recovery than primary surgeries, assessing the performance of UBE in this subset is of substantial clinical importance [9].

This study addresses this knowledge gap by evaluating the efficacy and safety of UBE in complex lumbar revision surgeries. It presents a consecutive series of 10 patients managed with UBE between February 2022 and February 2024. Clinical outcomes were analyzed using the Visual Analog Scale (VAS) for leg and back pain, the Oswestry Disability Index (ODI), complication rates, and patient satisfaction scores. By focusing on revision cases specifically, this study offers new insights into the potential of UBE to enhance visualization, minimize complications, and improve postoperative recovery in patients requiring reoperation. The findings contribute to the evolving body of evidence supporting UBE as a viable and advanced alternative to conventional open revision surgery.

## Materials and methods

This research was designed as a retrospective analysis of patients who underwent revision lumbar surgery utilizing the UBE technique. The study reviewed cases treated between February 2022 and February 2024 at a tertiary care center. The study population consisted of 10 patients who met specific inclusion criteria. To be eligible, patients must have had prior lumbar spine surgery and subsequently required revision surgery due to persistent or recurrent symptoms. Only those patients who underwent the revision surgery using the UBE technique and had complete clinical records and follow-up data were included in the study. The inclusion criteria for this study included patients with a history of previous lumbar spine surgery, patients requiring revision surgery due to unresolved or recurring symptoms, patients who underwent revision surgery via the UBE technique, and patients with comprehensive clinical records and follow-up information. The exclusion criteria included patients undergoing primary lumbar surgery, patients with incomplete clinical records or follow-up data, and patients who underwent revision surgery using alternative surgical techniques.

Patient data were meticulously gathered from electronic medical records. The collected data included demographic information such as age, sex, and body mass index; clinical history; details of previous surgeries; reasons for revision surgery; and specific surgical details regarding the UBE procedure. The UBE technique was conducted under general anesthesia with the patient positioned prone. Two small portals were established, one for the endoscope and another for the surgical instruments, and continuous irrigation was employed throughout the procedure to ensure a clear view of the surgical field. The surgery involved careful dissection of scar tissue, decompression of neural elements, and, when necessary, fusion or fixation using endoscopic instruments. Intraoperative neuromonitoring was utilized to enhance the safety and precision of the procedure.

Clinical outcomes were assessed using the VAS for leg and back pain and the ODI. Preoperative VAS and ODI scores were documented, and postoperative assessments were conducted at intervals of six months, one year, and two years. The intensity of pain was measured on a scale from 0 to 10, where 0 indicated no pain and 10 represented the most severe pain imaginable. Functional impairment was measured using the ODI, a validated tool that quantifies the impact of low back pain on daily activities, with scores ranging from 0 to 100 and higher scores reflecting greater disability. Data analysis was performed using statistical software such as IBM SPSS Statistics for Windows, Version 25 (Released 2017; IBM Corp., Armonk, New York). Descriptive statistics were used to summarize patient demographics and surgical details. Continuous variables were reported as means and standard deviations, while categorical variables were presented as frequencies and percentages. The primary outcomes focused on the changes in VAS and ODI scores from preoperative to postoperative assessments, and paired t-tests were employed to compare preoperative and postoperative scores, with a p-value of less than 0.05 considered statistically significant.

Complications were carefully documented and categorized into intraoperative and postoperative events. Intraoperative complications included issues such as dural tears, nerve injuries, and excessive bleeding, while postoperative complications covered infections, reoperations, and neurological deficits. Patient satisfaction was gauged during the final follow-up visit using a Likert scale ranging from 1 (very dissatisfied) to 5 (very satisfied). Patients were systematically followed up at six months, one year, and two years postoperatively. During these visits, clinical assessments, including VAS and ODI scores, were recorded, and any additional interventions or treatments required during the follow-up period were also documented.

## Results

The outcomes of 10 patients (five females, five males; mean age: 47.7 years) who underwent revision lumbar surgery using the UBE technique are summarized in Table 1. Previous surgeries included open, endoscopic, and microscopic discectomies as well as PLIF with unilateral screws, reflecting case complexity. Follow-up ranged from 6 to 24 months, with a mean of 13.2 months. Leg pain VAS scores improved from 5-9 preoperatively to 0-2 postoperatively, while back pain VAS scores decreased from 4-8 to ≤2. ODI scores improved from 40-75 to 10-40. Eight patients achieved excellent or good outcomes, while two had fair results, demonstrating the effectiveness of UBE in complex lumbar revision surgeries. Postoperatively, patients demonstrated substantial reductions in both leg and back pain, along with marked improvements in functional disability, as assessed by the VAS and the ODI. The average preoperative VAS score for leg pain, initially 8.1, showed a significant decline to 0.8 following surgery (p < 0.001), while the mean preoperative VAS score for back pain, which was 7.5, decreased to 1.7 postoperatively (p < 0.001). Regarding disability, the average preoperative ODI score of 67.5 significantly improved to 17.5 after surgery (p < 0.001).

**Table 1:** Patient demographics and baseline characteristics PLIF: Posterior Lumbar Interbody Fusion; VAS: Visual Analog Scale; ODI: Oswestry Disability Index

Case	Age	Gender	Initial Surgery	Follow-Up (Months)	Pre-op VAS (Leg)	Post-op VAS (Leg)	Pre-op VAS (Back)	Post-op VAS (Back)	Pre-op ODI	Post-op ODI	Outcome
1	52	Female	Open discectomy	6	9	0	8	1	70	10	Excellent
2	28	Male	Endoscopic discectomy	6	8	0	7	1	70	10	Excellent
3	49	Male	PLIF + unilateral screws	6	9	1	8	2	75	20	Good
4	38	Male	Microdiscectomy	24	9	1–2	8	2	75	15	Excellent
5	51	Female	Microscopic discectomy	24	8	0	7	1	70	10	Excellent
6	48	Female	Endoscopic discectomy	12	6	5–6	5	5	50	40	Fair
7	32	Male	Microscopic discectomy	12	8	0	7	1	70	10	Excellent
8	50	Female	Microscopic discectomy	12	5	4–5	4	4	40	30	Fair
9	64	Male	Microscopic discectomy	12	9	1	8	2	75	15	Excellent
10	45	Female	Open discectomy	12	9	1	8	2	75	20	Excellent

No major complications such as dural tears, infections, or nerve injuries were observed. Minor issues, including temporary leg pain and mild wound irritation, were rare and resolved without further intervention. Patient satisfaction was high, with seven patients reporting excellent outcomes accompanied by significant pain relief and functional gains, two reporting good outcomes with improvements despite some residual symptoms, and one reporting a fair outcome with moderate pain relief and limited functional improvement.

A 52-year-old woman who had previously undergone an open discectomy reported complete relief from leg pain and a substantial decrease in back pain, with her VAS scores improving from 9 to 0 for leg pain and from 8 to 1 for back pain, and her ODI score improving from 70 to 10. A 28-year-old man with a history of endoscopic discectomy experienced complete resolution of leg pain and a marked reduction in back pain, with his VAS scores improving from 8 to 0 for leg pain and from 7 to 1 for back pain, and an ODI improvement from 70 to 10 (Table 1).

**Figure 1:**
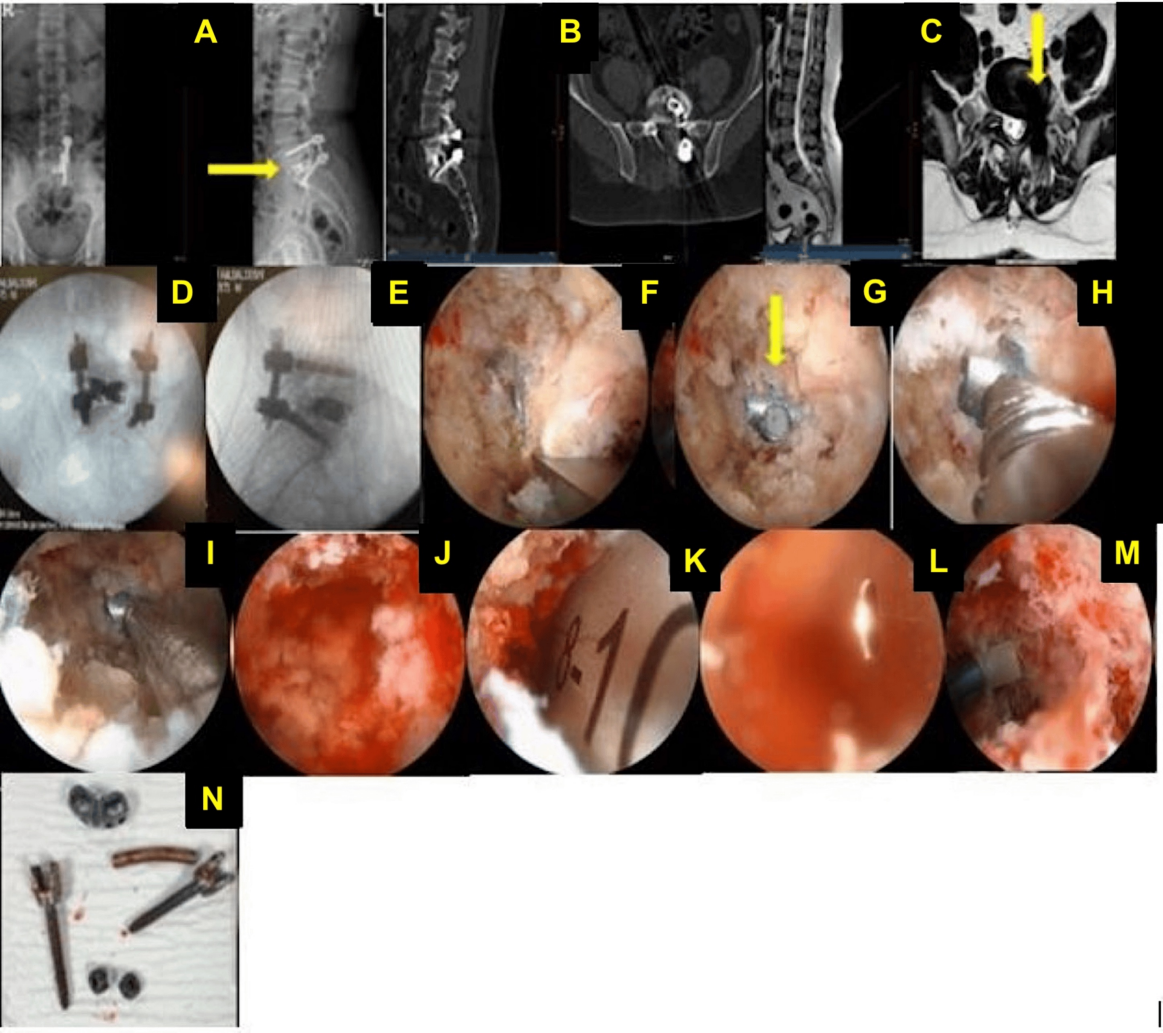
Lumbar spine revision surgery (A) Preoperative AP and lateral X-rays illustrate unilateral screw fixation on the left side at L5/S1, with the displaced cage (yellow arrow) causing compression of the thecal sac and nerve root. (B) Preoperative axial and sagittal CT scans displaying the displaced cage compressing the thecal sac and nerve root at L5/S1. (C) Axial and sagittal T2-weighted MRI images of the lumbar spine in a 49-year-old male presenting with cauda equina syndrome, showing severe compression at the L5/S1 level caused by cage displacement (yellow arrow) from a previous transforaminal lumbar interbody fusion (TLIF) surgery, compressing the thecal sac and nerve root. (D) AP intraoperative X-ray showing positioning of the cage and screws after left endoscopic removal of the malpositioned cage and screws, followed by left endoscopic TLIF using a dual-direction expandable cage. (E) Intraoperative image showing the endoscopic removal of an osteophyte during the procedure. (F) Intraoperative image showing the exposure of the previous TLIF cage. (G) Intraoperative image demonstrating an endoscopic trial connection of the cage holder to the previous TLIF cage. (yellow arrow). (H) Intraoperative image showing the successful connection of the cage holder to the previous TLIF cage. (J) Intraoperative image showing the endoscopic insertion of a cage trial during the procedure. (K) An intraoperative image showing the endoscopic insertion of a cage trial during the procedure. (L) An intraoperative image illustrating the endoscopic insertion of the new cage using a cage guide. (M) Intraoperative image capturing the endoscopic insertion of the new expandable cage. (N) Photograph of the removed cage and screws post-surgery.

**Figure 2:**
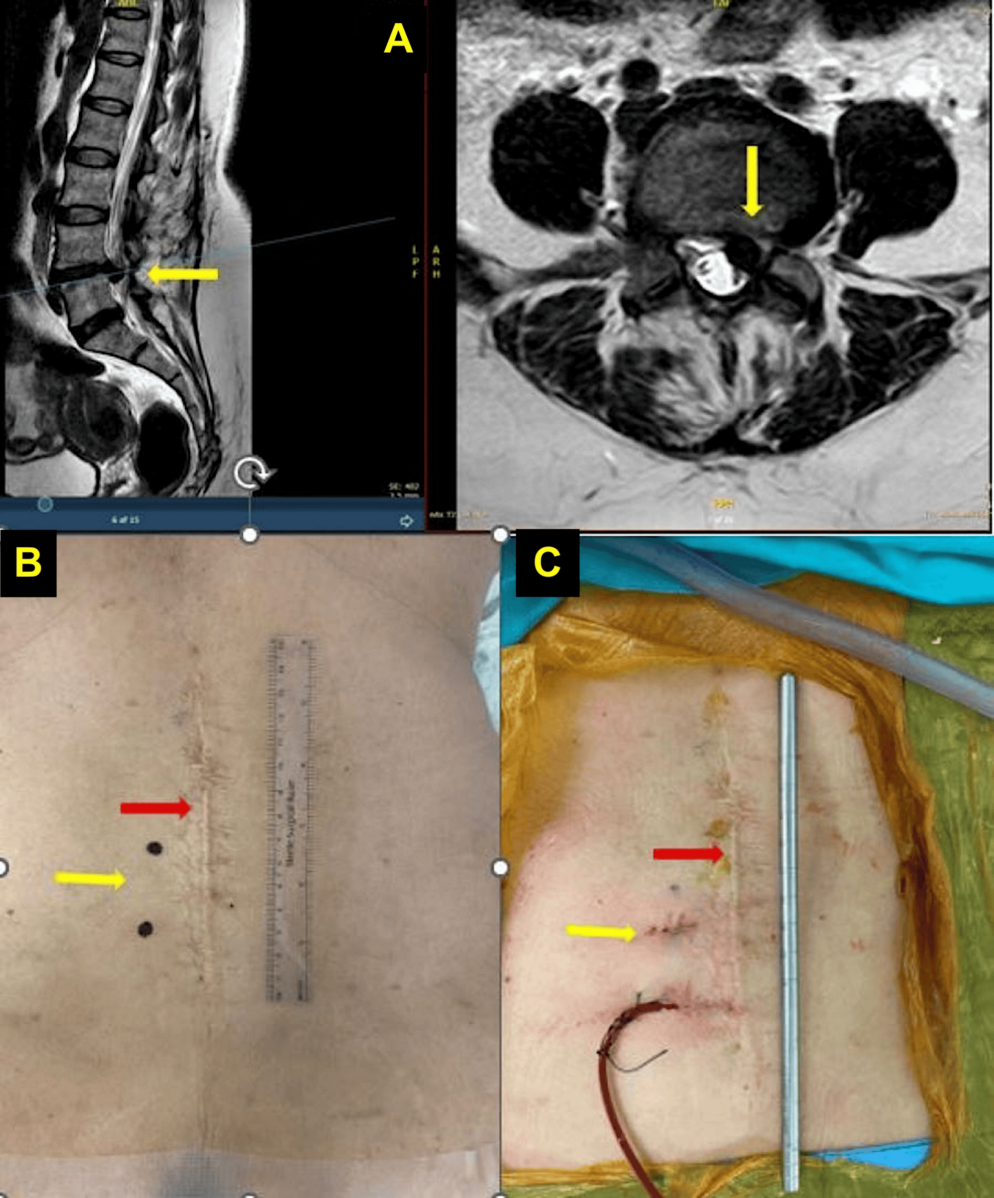
Ruptured disc at L4/L5 after 20 years post-discectomy (A) Axial and sagittal T2-weighted MRI scans of a 51-year-old female with a history of L4/L5 microscopic discectomy 20 years ago. The images reveal a recurrent disc herniation with a ruptured fragment impinging on neural structures (yellow arrow). This case highlights the long-term risk of re-herniation following lumbar disc surgery. (B) The back of a 52-year-old female with a large scar (red arrow) from an open L4/L5 discectomy done 20 years earlier. Small skin markers (yellow arrow) indicate the planned Unilateral Biportal Endoscopic (UBE) discectomy for recurrent symptoms, highlighting its minimal invasiveness compared to the original 15 cm incision. (C) The back of a 52-year-old female after a left UBE L4/L5 discectomy for recurrent symptoms. The image highlights the small UBE incisions (yellow arrow) compared to the larger scar (red arrow) from her prior open discectomy, demonstrating the reduced surgical footprint of the technique.

A 49-year-old man who had undergone PLIF with unilateral screws reported significant relief from pain, with VAS scores improving from 9 to 1 for leg pain and from 8 to 2 for back pain, while his ODI score improved from 75 to 20 (Figure 1). A 38-year-old man who had previously undergone microdiscectomy reported considerable pain relief, with VAS scores improving from 9 to 1-2 for leg pain and from 8 to 2 for back pain, and an ODI improvement from 75 to 15. A 51-year-old woman who had a microscopic discectomy experienced complete relief from leg pain and a significant reduction in back pain, with VAS scores improving from 8 to 0 for leg pain and from 7 to 1 for back pain, and an ODI improvement from 70 to 10 (Figure 2). A 48-year-old woman who had undergone endoscopic discectomy reported moderate pain improvement, with her VAS scores changing from 6 to 5-6 for leg pain and from 5 to 5 for back pain, while her ODI improved from 50 to 40.

A 32-year-old man who had previously undergone a microscopic discectomy experienced complete resolution of leg pain and a significant reduction in back pain, with VAS scores improving from 8 to 0 for leg pain and from 7 to 1 for back pain, and an ODI improvement from 70 to 10. A 50-year-old woman who had undergone microscopic discectomy reported moderate pain improvement, with VAS scores improving from 5 to 4-5 for leg pain and from 4 to 4 for back pain, and an ODI improvement from 40 to 30. A 64-year-old man with a history of microscopic discectomy experienced significant pain relief, with VAS scores improving from 9 to 1 for leg pain and from 8 to 2 for back pain, and an ODI improvement from 75 to 15. A 45-year-old woman who had undergone an open discectomy reported marked pain relief, with VAS scores improving from 9 to 1 for leg pain and from 8 to 2 for back pain, and an ODI improvement from 75 to 20.

The UBE technique proved effective in revision lumbar surgery, offering significant reductions in pain, improved functional outcomes, and high patient satisfaction. Complications were minimal, with no major adverse events, highlighting the safety and value of UBE as a minimally invasive option for complex lumbar revisions.

## Discussion

The UBE approach has demonstrated efficacy in revision lumbar surgery, resulting in significant reductions in VAS and ODI scores. By enhancing visualization, minimizing tissue trauma, and improving maneuverability, UBE effectively reduces pain and disability in complex surgical cases.

Compared with previous studies, the UBE approach shows outcomes that are equal to or superior to those of traditional open revision surgeries. Similar results with minimally invasive techniques were reported by Ertilav et al. (2023) and Liu et al. (2024), particularly in pain relief and functional recovery [10,11]. The reduced VAS scores in this study also align with Peng et al. (2019), who demonstrated the effectiveness of minimally invasive methods for pain control [12]. Achieving these results with minimal complications further supports UBE’s value in complex revision cases.

The UBE technique offers advantages such as reduced muscle dissection and blood loss, unlike traditional open revision surgeries that require extensive dissection and carry higher risks [4]. This study's findings showing VAS score reductions from 8.1 to 0.8 for leg pain and from 7.5 to 1.7 for back pain, with minimal complications, support UBE's effectiveness in relieving symptoms while minimizing tissue damage. The UBE technique's dual portal system enhances triangulation and instrument maneuverability, thereby improving precision compared with earlier minimally invasive methods. As noted by Xu et al. (2024), this is especially valuable in revision surgeries with altered anatomy, helping reduce risks like dural tears and nerve injuries common in open surgeries [13].

This study's low complication rate is notable compared with traditional open revisions, which carry a higher risk of dural tears, infections, and nerve injuries [14]. No major complications occurred, and minor issues like temporary leg pain and mild wound irritation were rare and resolved without further intervention, underscoring UBE's strong safety profile in complex revision cases.

High patient satisfaction further supports the success of the UBE technique. In this study, seven patients reported excellent outcomes with marked pain relief and functional improvement, while two reported fair outcomes. These results align with findings by Lee et al. (2023), who observed similar satisfaction levels in minimally invasive lumbar surgeries, emphasizing the patient-centered benefits of such approaches [15].

Long-term outcomes in revision lumbar surgeries are often less favorable than in primary cases due to altered anatomy [16]. However, this study, with follow-ups of 6-24 months, showed that UBE provided lasting pain relief and functional gains. The drop in ODI scores from 67.5 preoperatively to 17.5 postoperatively indicates sustained medium-term benefits.

The recurrence of symptoms in some patients highlights the need for vigilant follow-up. For example, a 51-year-old woman developed recurrent disc herniation 20 years after discectomy, reflecting the potential for long-term complications. This aligns with Park et al. (2023), who emphasized the importance of long-term monitoring to detect and manage late-onset issues [17]. Therefore, while UBE improves short-term outcomes, continued follow-up is vital to ensure lasting success and address late complications.

This study's findings have important implications for revision lumbar surgery. UBE, with its enhanced visualization, reduced tissue trauma, and greater precision, provides a strong alternative to open surgery, potentially lowering complication rates and improving outcomes. It is especially valuable in challenging cases such as recurrent disc herniations or hardware failures, where navigating scar tissue and altered anatomy with minimal additional damage is critical. The high patient satisfaction observed in this study suggests that UBE is effective both clinically and in meeting patient-centered care goals. As healthcare increasingly prioritizes outcomes and satisfaction, techniques like UBE that achieve both are likely to gain wider adoption.

Despite promising results, this study has limitations. The small sample size of 10 patients limits generalizability, and the retrospective design introduces potential biases, including selection and recall bias, which may affect validity. Larger, prospective studies are needed to confirm UBE's efficacy and safety in revision lumbar surgeries. Future research should focus on larger, prospective studies comparing UBE with other minimally invasive and open approaches to better define its benefits and limitations. Long-term follow-up is needed to evaluate durability, and studies on factors contributing to symptom recurrence or reoperation could help optimize techniques and improve outcomes.

## Conclusions

The UBE approach shows strong potential for revision lumbar surgery, providing notable improvements in pain and function with minimal complications. Its ability to address complex anatomy safely underscores its efficacy as a minimally invasive option. While the results are encouraging, further large-scale and long-term studies are needed to confirm these findings and define UBE's role as a standard of care in revision lumbar procedures.
